# How does the cerebellum contribute to cognitive functions?

**DOI:** 10.1371/journal.pbio.3003688

**Published:** 2026-03-23

**Authors:** Jörn Diedrichsen, Samuel D. McDougle

**Affiliations:** 1 Western Institute of Neuroscience, Western University, London, Ontario, Canada; 2 Department of Computer Science, Western University, London, Ontario, Canada; 3 Department of Statistical and Actuarial Sciences, Western University, London, Ontario, Canada; 4 Department of Psychology, Yale University, New Haven, Connecticut, United States of America; 5 Wu Tsai Institute, Yale University, New Haven, Connecticut, United States of America

## Abstract

Over the past 70 years, neuroscience has gained a deep understanding of how the cerebellum supports basic motor functions. Anatomical, clinical, and neuroimaging studies, however, have also firmly established that the cerebellum holds an important role in cognition. Even though this topic has received considerable attention, we still do not know the exact nature of this contribution. This Unsolved Mystery reviews known facts about how the cerebellum contributes to cognition and identifies roadblocks that have prevented the development of a unified theory. Addressing these key questions should help the field develop the testable, falsifiable hypotheses that are needed to solve this intriguing question.

## Abstract 2

The role of the cerebellum in motor functions is well understood. But why is the same circuitry engaged in functions such as working memory, language, and social cognition? This Unsolved Mystery looks at the problems that have made it so difficult to answer this question and outlines strategies to make progress.

## Introduction

The cerebellum is a brain structure full of contradictions. On the one hand, we know a lot about its anatomy, circuitry, and plasticity mechanisms. Indeed, for more than 50 years we have had a compelling theory of how the cerebellum learns, a theory that, in broad strokes, has stood the test of time remarkably well. Despite this wealth of insight, we still lack a concise answer to the seemingly simple question: what does the cerebellum actually do?

**Fig 1:**
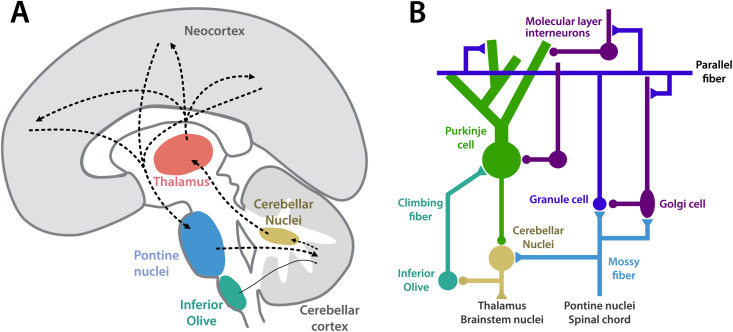
The cerebellar circuitry. **A.** Schematic diagram of the neocortico–cerebellar circuit, with pontine nuclei providing mossy fiber input, and the inferior olive providing climbing fiber input to the cerebellar cortex. The output is sent back to the neocortex via cerebellar nuclei and thalamus. **B.** Wiring diagram of the local circuitry within the cerebellum. Excitatory synapses are shown as triangles, inhibitory synapses as circles. We note that for simplicity this schematic is leaving out additional potentially important connections, such as projections from the cerebellar nuclei onto granule cells [3,4].

In the human brain, approximately 40 million axons leave the neocortex through the cerebral peduncles [1], and most of these send collaterals to the pontine nuclei (Fig 1A), which then give rise to mossy fibers (Fig 1B). In the cerebellar cortex, these mossy fibers synapse onto 50 billion granule cells, which make up more than half the neurons in the human brain [2]. The axons of granule cells, the parallel fibers, then connect to Purkinje cells (Fig 1B), the output neurons of the cerebellar cortex. In the human, each Purkinje cell receives ~1,000,000 parallel fiber synapses. Purkinje cells have a high spontaneous firing rate (50–70 Hz) and tonically inhibit the downstream cerebellar nuclei.

Purkinje cells are also innervated by one, or sometime two [5] climbing fibers, which originate in the inferior olive and which fire at relatively low rates (0–3 Hz). Despite its relative sparseness, the climbing fiber input acts as a strong plasticity signal that modifies parallel-fiber-to-Purkinje cell synapses [6]. This and other plasticity sites create a circuit, in which each Purkinje cell learns to predict its climbing fiber input from the concurrent high-dimensional activity patterns of its parallel fibers. The firing rate of the Purkinje cell then goes down, releasing the inhibition of the deep cerebellar nuclei cells, which start to fire vigorously. The prediction is usually well-timed, anticipating the climbing fiber input by tens to hundreds of milliseconds. Cells in the cerebellar nuclei then project back to a range of structures: the inferior olive, other subcortical nuclei, the cerebellar cortex itself [3], and, most prevalent in the human, via the thalamus to the neocortex. In summary, the cerebellum looks like a high-capacity learning engine that can provide a precisely timed predictive signal learned from a very high-dimensional input.

This basic idea of how the local cerebellar circuit learns and predicts was formulated by Marr [7], Albus [8], and Ito [9], and has evolved into a well-established theory [10]. Among other functions, this framework can successfully explain many of the basic phenomena seen in cerebellar contributions to sensorimotor tasks such as eye-blink conditioning [11], adaptation of the vestibular-ocular reflex, and modulation of smooth-pursuit eye movements [12,13].

## Cerebellar function in cognition

**Fig 2:**
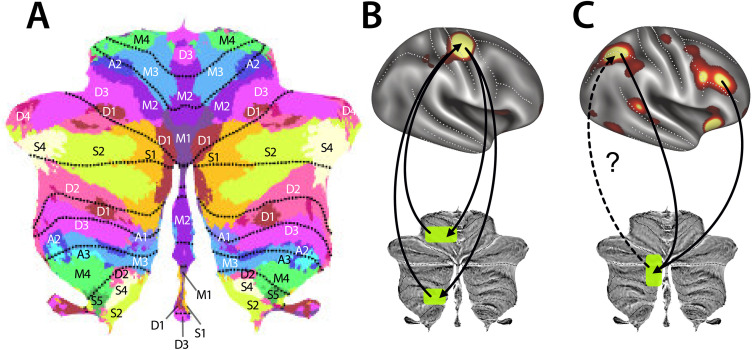
Cerebellar functional areas and loops. **A.** Functional parcellation displayed on a flattened representation of the human cerebellar cortex [27]. The color assigned to each parcel is a representation of the activation profile across many different cognitive and motor tasks. Green and bluish areas activate for movements of different body parts (M1–M4) and for action observation (A1–A3), red areas (D1–D4) for working memory and executive function, and yellow areas (S1–S5) for social and language functions. **B.** Closed-loop connectivity between the hand area of primary motor cortex (M1) and the superior and inferior hand motor region (M3) in the contralateral cerebellum. **C.** Convergence of frontal and parietal cortical areas onto a spatial working memory region (D1) in the contralateral cerebellum. From functional MRI analysis it remains unclear which cortical areas this region projects back to (dashed line).

Even though the most salient symptoms of cerebellar damage or degeneration in adulthood are the disruption of the smooth coordination of movement [14], the majority of the human cerebellum is likely not concerned with motor control but instead contributes to a wide range of cognitive functions. Leiner, Leiner, and Dow [15] first suggested that the disproportional expansion of the lateral cerebellum and the dentate cerebellar nuclei in human brain evolution (see Box 1) is due to its contributions to cognition. Since then, it has been shown that many cerebellar regions receive input from [16] and deliver output to [17,18] non-motor areas in the parietal, prefrontal, temporal, and parahippocampal cortices. Indeed, it has been argued that the different cerebellar regions form closed and largely separated loops with many neocortical areas that are not directly implicated in motor control [19]. Consistent with these anatomical observations, patients with cerebellar damage sometimes do not demonstrate significant motor deficits, but instead (or additionally) exhibit a range of cognitive symptoms [20–22] that, while often more subtle than motor problems, are nonetheless replicable and robust. Moreover, functional neuroimaging studies have shown that the cerebellum reliably activates during most cognitive tasks [23–26]. Systematic mapping studies have revealed a detailed map of the functional specialization of the human cerebellum (Fig 2A), with different subregions engaged in functions such as action observation, verbal and spatial working memory, executive functioning, language, social cognition, and even imagination [27].

**Box 1. What do seals, elephants, bats, crows, and humans have in common?**

The genetic blueprint for a fully developed cerebellum appeared first in jawed fish and is present in all vertebrates descending from this common ancestor [28]. From then on, there was a tendency for the cerebellum to enlarge in proportion with the rest of the brain. However, for some species the entire cerebellum has enlarged to an unusual degree, for example for weakly electric fishes (Mormyridae [29]), elephants [30], bats [31], and crows and other large-brained birds [31]. Other species show a specific enlargement of the cerebellar hemispheres over the vermis, for example in seals (pinnipeds), dolphins (cetaceans [32]), and apes (including humans [33]). Importantly, these adaptations cannot be traced to a common ancestor but appear on unconnected branches of the evolutionary tree. This suggests that at multiple times in evolutionary history there existed selective pressures for which a large cerebellum provided a competitive solution and increased fitness. What problem the cerebellar circuit was able to solve in each case appears to vary widely across the animal kingdom, and includes functions such as electric sensing, echolocation, vocal learning, complex social behavior, control of a flexible trunk, skilled object manipulation (with a beak or hand), and, especially in humans, higher cognitive functions. Why did these functions (and not others) depend on a large cerebellum? For example, what is special about the control of a flexible trunk that has led to cerebellar enlargement in elephants and weakly electric (or elephant) fish in parallel? How did the octopus solve a similar biomechanical problem of controlling its arms without a cerebellum? Studies of distantly related animals that show similar behavioral adaptations associated with the use of increased cerebellar territory may provide insight into the computational characteristics of problems that the cerebellar circuit is especially good at solving. From evolutionary history, we know that the answer to this question cannot be a simple one and must account for an astounding variety of behaviors.

But how does the cerebellum contribute to all these disparate cognitive functions? One enduring mystery is whether the cerebellum performs algorithmically similar computations across motor and cognitive domains (a so-called “universal cerebellar transform”), or whether it has distinct computational roles across different domains. Moreover, linking these algorithmic-level questions to the circuit architecture of the cerebellum remains a difficult but critical task.

## A universal cerebellar transform?

The local micro-circuit of the cerebellar cortex is, relative to the neocortex, quite homogenous across functional regions. This has led to the tempting idea that the cerebellum performs a uniform computational function within each cortico–cerebellar loop. In essence, it has been suggested that cognitive areas in the cerebellum modulate the activity of cortical association areas to allow for ‘coordinated’ cognitive processes, in the same way that the motor areas of the cerebellum modulate activity in primary motor cortex to allow for coordinated movements [34].

Despite the intuitive appeal of this idea, very little progress has been made to identify what this universal function may be. The main hurdle has been to develop theories that are formalized concretely enough to generate testable empirical predictions. In other brain regions, such as the hippocampus, formal functional theories are starting to be developed. For example, the computational framework of grid cell coding has been successfully applied not only to navigation tasks in physical spaces, but also to cognitive tasks in conceptual spaces [35,36].

Why has it been so difficult to achieve something similar for the cerebellar circuit? One possible answer is that the question, “what is the function of the cerebellum?,” does not have a more concrete answer than the question, “what is the function of the neocortex?.” Indeed, there is increasing evidence for differentiation in the microcircuitry across the cerebellum [37]. Furthermore, different cerebellar regions interact with cortical areas using potentially different patterns of connectivity, such that the way the cerebellum influences cortical function may be quite different across different cortico–cerebellar loops [38,39].

If this is true, then a more productive approach may be to investigate the contribution of the cerebellum in each of the cortico-cerebellar loops separately first, without *a priori* assuming that it serves the same function as in the neighboring loop. If there truly is a shared computational principle that characterizes the cerebellar contribution across all these loops, it will emerge with the data. In the remainder of this Unsolved Mystery, we attempt to outline what specific questions need to be resolved, for each cortico-cerebellar loop, in order to make concrete progress in characterizing cerebellar function.

## How is the cerebellum connected to other brain regions?

If the basic element of our investigation is the cortico-cerebellar loop, then we first need to identify them. In the rodent and monkey brain, viral tracing techniques [18] are able to precisely map multi-synaptic connections. Since these techniques are not available in the human brain, most of what we know about cortico-cerebellar connectivity has been indirectly inferred from the correlations of functional magnetic resonance imaging (fMRI) signals between the neocortex and the cerebellum. Most studies of this type are based on resting-state data, starting with the seminal work by Buckner and colleagues [40]. After subdividing the neocortex into distinct resting-state networks, the authors generated a functional map of the cerebellum by assigning each cerebellar voxel to the cortical network it was most correlated with. Later work replicated the main features of this basic connectivity pattern using both resting-state [41,42] and task-based activity maps [27,43]. Given that variations in the cerebellar fMRI signal likely reflect mostly mossy fiber inputs (and local processing of those inputs in the granule cell layer), but not the activity of the output of the cerebellar cortex [44], we hypothesize that fMRI correlations are predominantly shaped by projections from neocortex to cerebellum, rather than revealing much about the projections from the cerebellum back to the neocortex.

Across different connectivity models, several clear insights have emerged. First, most of the cerebellar input arises from the contralateral cerebral hemisphere, consistent with the crossing of mossy fibers originating from the pontine nuclei, with a more modest degree of uncrossed input. Second, while nearly all cortical areas appear to be functionally connected to the cerebellum, there are considerable differences in how much of the cerebellum versus the neocortex is occupied by each cortico-cerebellar loop. For example, the cerebellar contributions to visual networks appear to be disproportionally small, whereas the size of the regions dedicated to executive functions (i.e., the fronto-parietal network) is disproportionally large [27,40,41]. Surprisingly, the size of motor-related regions in the cerebellum roughly matches their relative size in the neocortex. Third, a single cortical area often connects to multiple spatially non-contiguous regions of the cerebellum; for example, the hand area of primary motor cortex is connected both with the superior (lobules V, VI) and inferior (lobules VIII) hand representation in the cerebellum (M3; Fig 2B), and the default-mode network appears to be connected to three distinct sub-regions in the cerebellum (S3 in crus I, crus II, lobule IX) [27,45,46]. Finally, it has been suggested that there is substantial convergence of multiple cortical areas onto the same cerebellar region, and that this convergence is especially pronounced in the so-called ‘cognitive areas’ of crus I and crus II (Fig 2C) [43]. There is also evidence from tracing studies in the mouse that suggest that each cerebellar area projects back to multiple cortical areas [47]. It is therefore possible that the synchronized cerebellar input to these neocortical regions changes the coherence of neuronal activity between them [48]. In this way, the cerebellum may help to coordinate the communication between distal pairs of cortical regions, rather than fine-tuning neural dynamics within a single region.

Accurate connectivity models between the cerebellum and the rest of the brain are an essential tool if we want to understand the role of the cerebellum across domains, as these models tell us which brain regions provide input to—and receive output from—each specific cerebellar area. This knowledge is essential, because it allows us to analyze the neural activity in each cerebellar region in the context of the neural activity of other regions within the same cortico-cerebellar loop. Time delays and changes of representations can provide insights into the computations that occur at each stage of the loop. For functional imaging in humans, we now have task-invariant connectivity models that make quantitative and testable predictions about the amount and exact pattern of cerebellar activity, based solely on the neocortical data for same tasks [27,43]. Deviations from such predictions indicate that the cerebellar fMRI activity is not just a linear function of its inputs, but rather suggest task-dependent gating or transformation of those inputs, providing potentially critical insights into specific cerebellar functions [49].

## How is information coded in the granule cell layer?

One special characteristic of the cerebellar circuit is the massive information expansion in the granule cell layer. Each single mossy fiber contacts tens to hundreds of granule cells, and each granule cell integrates input from 4–5 mossy fibers, often coming from different sources. Even if the mossy fiber to granule cell connectivity was entirely random, the vast numbers of granule cells and the diversity of synaptic characteristics [50] ensure a powerful non-linear expansion of the original input, which is well-suited for learning complex functions [51,52]—that is, for performing pattern separation. This feature was the central tenet of Marr’s original formulation of why the cerebellum may be a powerful learning machine, and has been further developed in recent papers [53,54].

Given that it is difficult to record from isolated granule cells (which are very small and tightly packed), direct tests of this idea have been missing until very recently. While an initial study showed negative findings [55], we are only now seeing the first direct evidence that the granule cell layer may indeed perform a computation akin to non-linear function expansion. These recent results indicate that the granule cell layer code is indeed high-dimensional [56], and that it is sparse while also combining information from multiple modalities in a non-linear fashion [57]. Nonetheless, the exact characteristics of the granule cell population code are only now beginning to be revealed.

What might the cerebellar information expansion achieve for cognitive functions? One possibility is that information processing in the mossy fiber layer is especially useful to learn precise non-linear functions of time using a rich set of temporal basis functions [53,58]. This has been extensively demonstrated in basic sensorimotor tasks, such as eyeblink conditioning. In this paradigm, the conditioned stimulus (e.g., auditory tone) activates the mossy fiber, followed by an unconditioned stimulus (an air puff to the eye), which in turn activates climbing fibers. In this context, the granule cell layer creates a distributed temporal code through the diverse response dynamics of individual granule cells. This allows Purkinje cells to learn the exact parallel fiber pattern that precedes the climbing fiber input, thereby building up a temporally precise prediction of when the air puff will occur [54].

Where in the cognitive domain do temporally precise predictions matter? While many cognitive processes seem to occur at slower timescales, there are examples in language comprehension and during social interactions, where the exact timing of stimuli matters. For example, genuine smiles are returned with a median latency of 750ms [59], and it is possible that the cerebellum is involved in the production and perception of such precisely timed behaviors. To test this idea, characterizing the importance of the temporal dimension across cognitive and social tasks and probing the cerebellar involvement in them will be the next critical step. It should also be noted, however, that some behaviors that rely on the cerebellar circuit do not seem to require accurate timing [60–62], suggesting that non-linear function expansion, if it is a critical feature of cerebellar computation, may also be used for non-temporal information.

## What information is carried by climbing fibers in cognitive tasks?

Climbing fibers provide the main teaching signals that shape the output of the cerebellar cortex—the firing rate of Purkinje cells. Across different sensorimotor tasks, it has been important to understand what information climbing fibers convey, as it provides insights about what the cerebellar circuit tries to learn or predict. In most motor tasks, climbing fibers appear to convey information about motor errors, which the cerebellar learning mechanism can then help to compensate for. What do we know about climbing fiber signals in cognitive tasks?

Recent work in reward learning tasks has greatly expanded traditional conceptions of climbing fiber signals. In one influential study, Heffley and colleagues [63] designed a task that required rodents to learn, via reward feedback, novel sensorimotor associations between abstract visual stimuli and actions. Climbing fibers appeared to convey task-specific predictions about reward outcomes rather than signaling motor errors. The types of climbing fiber signals were diverse, reflecting events like reward prediction errors, unexpected rewards, and reward omissions. These and similar findings, both in rodents and non-human primates, suggest that cerebellar climbing fibers flexibly encode abstract, task-specific variables and contingencies, not only motor errors.

Furthermore, climbing fiber signals often convey that a surprising outcome occurred, but not whether that outcome was unexpectedly rewarding or non-rewarding. Furthermore, climbing fibers did not always distinguish between the sensory cues that differentially predict reward, even when the animal differentiates those cues behaviorally [64]. Therefore, these signals do not fit well with the notion that climbing fibers carry a specific and signed prediction error that can be used as a teaching signal for supervised learning. Thus, further work is needed to better understand the role of climbing fiber signals, by studying a wider range of cognitive tasks in rodents and non-human primates.

However, some cerebellar functional domains, such as language [65], will be difficult or impossible to study in animal models. In these cases, it is even less clear what information is carried by climbing fibers. Therefore, it will also be essential to develop improved techniques for measuring inferior olive activity using non-invasive methods in humans. Despite a few encouraging reports [66–68], fMRI of the inferior olive remains extremely difficult given the reduced signal-to-noise ratio, spatial distortions, and artifacts induced by the cardiac cycle [69]. Solving these problems and demonstrating reliable, spatially specific activity within the inferior olive across different domains would be a great step forward in understanding the role of the cerebellum in cognitive tasks. The nature of the climbing fiber input will inform us about what the cerebellar circuitry is trying to learn.

## How does the cerebellum modulate neural dynamics in the neocortex?

Cerebellar output contributes to cognitive functions by modulating the dynamics of recurrent activity in the neocortex via modulation of the thalamus. If there is a common principle of cerebellar function across domains, it must be found in how it affects thalamo-cortical activity dynamics. To glean insights into this process, several labs have started to apply temporally precise perturbations of cerebellar activity while measuring the resulting influence on behavior and cortical activity. For example, a study in mice [70] showed that cerebellar output to the anterior lateral motor area (a neocortical premotor structure) is essential for sustaining the preparatory neural signals associated with motor planning. Delay period motor planning could be causally disrupted by perturbing cerebellar output without interfering with the execution of movements. Similar results were also found in a task that required the accumulation of sensory evidence to guide perceptual decision-making [71]. These results indicate that cerebellar output may be important for the maintenance and dynamic updating of neocortical representations of abstract internal goals or decision variables.

Superficially, a role in maintaining cortical representations that evolve at a relatively slow time scale does not fit well with the idea that the cerebellum is critical for regulating the precise temporal dynamics necessary for many motor behaviors [53,54]. Observing the influence of cerebellar disruption on thalamo-cortical activity across a wider range of tasks will be critical for identifying whether there is a common dynamic motive across cortico—cerebellar loops, or if the temporal constraints on cerebellar contributions are in fact looser than commonly believed.

## What is the cerebellum’s role in cognitive development?

Perinatal lesions to the cerebellum have a profound impact on motor, language, and cognitive development [72,73] and substantially increase the likelihood of an autism diagnosis [74]. The resection of parts of the cerebellum during childhood can cause a complete or partial cessation of speech, a symptom called cerebellar mutism [75]. Importantly, equivalent lesions in adulthood do not cause similar symptoms, or if they do, lead to much milder deficits. These facts contrast directly with what is observed for lesions of the neocortex; for example, early lesions to language-related regions can lead to substantial reorganization and recovery of function, whereas the same lesion in adulthood leads to lasting deficits.

These observations have led to the idea that the cerebellum helps to ‘set up’ neocortical circuits underlying cognition in development [74]. If the cerebellum is lesioned during this critical period, development is delayed; however, once the neocortical regions have been established, the same cerebellar circuit may not be necessary anymore. This idea has also been extended to the aging brain. Here the cerebellum may have a neuroprotective role; for example, it may help to reorganize cortical circuits to compensate for tissue loss in the early stages of dementia [76].

While compelling, this idea is unlikely to provide a complete characterization of cerebellar function in general. Lesions to cerebellar motor areas lead to profound ataxia both in children [75] and adults, indicating that the cerebellum sometimes provides functions that cannot easily be replaced by a well-trained neocortex.

## Conclusions and future directions

Despite a wealth of knowledge about the cerebellar circuit itself, a general theory of how the cerebellum contributes to both cognition and motor control has remained elusive. In this Unsolved Mystery, we argue that cerebellar output may contribute to function in different ways depending on the system it is embedded in. This makes it necessary to study different cortico-cerebellar loops, especially those involved in cognitive function, without the *a priori* assumption that the results will generalize across loops directly.

Taking a systems-level view also implies that it will be difficult to understand the contributions of the cerebellum to cognition before we have better models of how cognitive functions arise from the neuronal dynamics across different cortical regions. A possible recipe for scientific progress involves three necessary steps. First, for a cerebellar region of interest, we need to characterize the sources of inputs and the targets of outputs, both in the thalamus and neocortex. Second, it will be important to record activity in as many brain areas in this loop as possible and see how information is manipulated throughout the loop and how it relates to the behavior of interest. Third, targeted perturbations in selected structures with simultaneous recordings in others will provide the critical data to disambiguate between different network models. While this all sounds extremely ambitious, technological advances in recording and stimulation are finally bringing such studies into reach [77].

Given these new data, what will a theory of cerebellar function in cognition finally look like? One key element will be to build biologically constrained computational models of cerebellar circuit function (for a recent example, see [49]). These models may be built by fitting neural data from a sensorimotor task, but the cerebellar circuit could then be recombined with a model of cortical association areas. It will be enlightening to see to what degree the function of the same circuitry changes when embedded in a system with different task representations and neural dynamics. Ultimately, such models should be able make specific predictions about the influence (or non-influence) of cerebellar disruptions onto cognitive tasks that can then be tested directly. Theory driven studies like this would be one sign of significant progress.

## Abbreviation:

fMRI: functional magnetic resonance imaging
